# Tai Chi exercise of short duration alters circulating oxylipins in postmenopausal women: a pilot study

**DOI:** 10.3389/fmed.2026.1907924

**Published:** 2026-08-31

**Authors:** Chwan-Li Shen, John W. Newman, Isabel F. Snodgrass, Danielle J. Caruso, Ming-Chien Chyu, Volker Neugebauer, Bruce A. Watkins

**Affiliations:** 1 Department of Pathology, Texas Tech University Health Sciences Center, Lubbock, TX, United States; 2 Center of Excellence for Integrative Health, Texas Tech University Health Sciences Center, Lubbock, TX, United States; 3 Center of Excellence for Translational Neuroscience and Therapeutics, Texas Tech University Health Sciences Center, Lubbock, TX, United States; 4 USDA, Agricultural Research Service, Western Human Nutrition Research Center, Davis, CA, United States; 5 Department of Nutrition, University of California, Davis, CA, United States; 6 West Coast Metabolomics Center, Genome Center, University of California, Davis, CA, United States; 7 Department of Medical Engineering, Texas Tech University, Lubbock, TX, United States; 8 Department of Pharmacology and Neuroscience, Texas Tech University Health Sciences Center, Lubbock, TX, United States; 9 Garrison Institute on Aging, Texas Tech University Health Sciences Center, Lubbock, TX, United States

**Keywords:** endocannabinoid-like compounds, endocannabinoids, fatty acids, oxylipins, postmenopausal women, Tai Chi

## Abstract

Tai Chi (TC) is a low-impact physical activity known to improve balance, strength, and cognitive health across the lifespan. We previously reported that 8 weeks of TC altered brain connectivity and circulating oxylipins (OxLs) and endocannabinoids/endocannabinoid-like compounds (eCBs) in postmenopausal (PM) women with knee osteoarthritis. This pilot study examined whether a short TC intervention similarly affects inflammatory OxL and eCB in PM women. Participants completed four TC sessions on non-consecutive days over 10 days. Each session included 10 min of warm-up, 45 min of 24-form Yang-style TC (six repetitions at ~7 min per routine), and 5 min of cool-down. Non-fasting plasma collected at baseline and immediately after the fourth session was analyzed for OxL and eCB using targeted metabolomics. Partial least squares discriminant analysis (PLS-DA) assessed pre-to post-intervention differences, and paired *t*-tests with false discovery rate correction evaluated metabolite changes. Sixteen PM women (58.9 ± 5.2 years; BMI 33.6 ± 5.4 kg/m^2^) completed the study. Several OxLs demonstrated PLS-DA variable importance in projection scores >1.0. Post-intervention, 1-AG and 12-hydroxyeicosatetraenoic acid (12-HETE) were generally lower, whereas 18-HEPE was higher. TC produced modest effects on eCBs but more pronounced changes in OxLs. Reduced monoacylglycerols may indicate TC-induced mobilization of fatty acids through enzymes involved in 1-AG metabolism, including phospholipase C, diacylglycerol lipase-α, monoglyceride lipase, and potentially fatty acid amide hydrolase.

## 1 Introduction

The endocannabinoid system (ECS) is comprised of G-protein-coupled cannabinoid receptors CB1R (type1) and CB2R (type 2), and endogenous arachidonic acid-derived canonical ligands arachidonoyl ethanolamide (AEA, i.e. anandamide) and 2-arachidonoyl glycerol (2-AG). Modern analytical techniques are often able to measure these species, along with a variety of chemically similar, yet CBR-inactive acyl ethanolamindes and acyl aminoacids, the endocannabinoid-like compounds. The ECS and its receptors are widely found in the central and peripheral nervous system, the immune system (1, 2), as well as in a broad array of peripheral organs and tissues like muscle and adipose (3). CB receptors are endogenously activated by AEA and 2-AG and inactivated through a membrane transporter facilitated re-uptake. The fatty acid ethanolamides are degraded by an intracellular fatty acid amide hydrolase, while 2-AG is degraded enzymatically by monoacylglycerol lipase (MAGL) 2-AG (2). 2-AG signaling is also dampened by the spontaneous acyl-migration from the *sn1* to *sn2* position, to form the more stable and less active 1-AG (4). Endocannabinoid (eCB) signaling acts through autocrine and paracrine stimulation of its receptors in neuropathic pain and neuroinflammation (5), and an increase in eCB receptors can induce neuropathic pain and neuroinflammation (5). The ECS also has roles in appetite, macronutrient metabolism (6), pain sensation, control of chronic pain, mood/mood disorders, and regulation of immune cell functions (7, 8).

Aerobic exercise (e.g., hiking and running) has shown to increase blood levels of AEA and 2-AG (9, 10). However, it appears that exercise intensity plays a bigger role than exercise mode in the increase in AEA and 2-AG concentration. Moreover, moderate exercise improves cognition, memory, and wellbeing in adults (11, 12). Women appear to have greater increases in AEA compared to men post-run; however, regular runners experienced greater mood increase and better post-run than occasional runners as assessed by Weiermair et al. (13). Exercisers experience decreased tension, depression, and anger via an exercise induced brain-derived neurotrophic factor (BDNF), a possible molecule involved in brain plasticity (14). BDNF may be associated with the anti-depressant effects of exercise, resulting from elevated AEA and eCB signaling in young male cyclists (15). Low levels of moderate exercise are positively associated with plasma anandamide (AEA, arachidonate form of eCB) levels and reduced anxiety (16). Research suggests a likely relationship for exercise to improve wellbeing via the ECS and actions on BDNF (7, 17).

Chronic or repeated stress results in a prolonged elevation of cortisol, the endogenous glucocorticoid stress hormone, via the hypothalamic-pituitary-adrenocortical axis whereas exercise seems to improve neurobiological conditions via the ECS (16). Tai Chi (TC) is a mind-body moderate intensity exercise shown to improve pain and reduce inflammation in subjects (18, 19). Being a moderate intensity exercise, TC may reduce chronic stress via eCB signaling in adults and older adults (7). We previously reported differences in circulating concentrations of oxidized lipid (OxLs) mediators of inflammation and eCBs among postmenopausal (PM) women with knee osteoarthritis (OA) after 8 weeks of pre- and post-TC (18, 19). However, it is unclear if fewer TC exercise sessions can alter OxL and eCB concentrations in healthy individuals.

Our research objective is to determine if circulating levels of eCB and OxL are changed after conditioning TC exercise for 4 sessions compared to baseline (before TC exercise). We hypothesize that four sessions of TC exercise (during an exercise period over 10 d) would increase 2-AG levels and reduce pro-inflammatory OxL levels in PM women.

## 2 Methods

Because this was an exploratory pilot study, no formal *a priori* power analysis was conducted; the study was intended to provide feasibility data and preliminary effect size estimates for future larger trials. This study was a pre-post study design including self-reported PM women completed 4 TC sessions. Each session was 60 min long. A total of two non-fasted blood samples were collected from each participant: one immediately before the first TC session (baseline) and the other immediately after completion of the fourth TC session. Blood was drawn into BD Vacutainer^®^ green-top tubes containing lithium heparin and kept at room temperature for 20 min before centrifugation at 2,500 × *g* for 10 min at room temperature. Following centrifugation, plasma was separated and stored at −80 °C until analysis of eCBs, OxLs, and PUFAs. It should be noted that isomerization and interconversion of 2-acylglycerols to 1-acylglcerols can occur at room temperature under the preanalytical time frame experienced by the samples (20). While this likely influenced the measured concentration of these species, the relative abundance of 2-AG to 1-AG were highly correlated in samples (*r* = 0.86, *p* < 0.0001), suggesting that preanalytical isomerization was consistent across the collected study samples. However, as steps were not taken upon sample collection to prevent MAG isomerization or degradation, the reported concentrations may correlate with but likely do not represent the actual endogenous concentrations or distributions of the MAGs reported.

This study was approved by the Institutional Review Board at the Texas Tech University Health Sciences Center (IRB #: L19-055) (ClinicalTrials.gov Identifier: NCT03823157).

### 2.1 Subject recruitment

Self-reported PM women, irrespective of ethnicity/race, were recruited for this study through the following: (1) recruiting participants from clinics at Texas Tech University Health Sciences Center (TTUHSC) (i.e., Family & Community Medicine, Internal Medicine); (2) placing printed advertisements in local newspapers and community centers; (3) distributing flyers at local outpatient clinical sites; (4) promoting study at local radio, institutional announcement, health fairs, and TV (i.e., KCBD Healthwise); (5) recruiting participants who have given written permission via informed consent on previous studies to be contacted for future research studies and/or who have submitted their information to be contacted for research participation on the secure TTUHSC Clinical Research Institute Volunteer Database; and (6) posting flyers on TTUHSC bulletin boards. Interested volunteers were instructed to call/text or email a study designated phone/email account. Study staff asked initial screening questions, and if eligible, the volunteer was scheduled for an informed consent to sign up for consent and HIPAA forms, and to complete a detailed medical questionnaire.

Inclusion criteria included: (1) Postmenopausal women (menopause is defined as the absence of menstrual period for 12 months, not related to pregnancy/lactation, hormonal contraception); (2) Age 40–70 years old; (3) BMI between 25–35 kg/m^2^; (4) English literacy. Exclusion criteria included: (1) Prior experience with mind-body practice (e.g., TC, Qi Gong, yoga, meditation) within 3 months prior to enrollment; (2) Severe medical limitations (i.e., dementia, symptomatic heart or vascular disease, or recent stroke) precluding full participation; (3) Medical/neurologic or other systemic diseases affecting the musculoskeletal systems (i.e. polio/Parkinson's/ multiple sclerosis, etc. in addition to cerebral vascular accident or stroke) and diabetes with peripheral neuropathy affecting their sensory/balance; (4) taking prescription pain/anti-inflammatory and/ or prescription sleep medications within the 3 months before study starts. Characteristics of the study population are shown in Table 1.

**Table 1:** Demographic characteristics of study population.

Variables	Units	
Subjects	n	16
Age	y	58.9 ± 5.2
Weight	kg	81.1 ± 13.9
Height	cm	155 ± 7
Body mass index	kg/m^2^	33.6 ± 5.4
**Medical history questions**		**Number/(%)**
General health rated “good”		15 (93.8)
Hormone or hormone–like therapy use		2 (12.5)
Prior mind–body exercise experience (> 3 months before enrollment)		2 (12.5)
Regular physical activities		11 (68.8)
History of stroke		1 (6.3)
History of osteoarthritis with no pain med		2 (12.5)
History of fibromyalgia with no pain med		1 (6.3)
History of low back pain with no pain med		2 (12.5)
History of steroids use (> 3 months before enrollment)		2 (12.5)
History of cigarettes (current or ever–smoked)		2 (12.5)
Alcohol consumption		7 (43.8)

All data are mean ± standard deviation unless otherwise specified.

### 2.2 Tai Chi intervention

There were only four sessions of TC on non-consecutive days but performed within a 10 d window. The 24-form Yang style TC was employed in this study. The TC intervention was led by a TC master with 25-yr TC practice and teaching experience. In each session, in addition to 10 min of warm-up and 5 min of cool-down exercises, the routine of the 24-form Yang style TC was repeated 6 times during the 45 min training period based on the standard speed of ~7 min per routine (21). The 24-form TC consisting of 24 movements of martial application performed slowly and gently while breathing deeply and meditating. A detailed TC program manual containing pictures and a description of how to perform each pose with typical instructor dialogue was developed for this study. Classes typically involved a review of TC principles and movement, breathing techniques, and relaxation methods. The instructor explained and demonstrated how the exercise should be performed, and subjects followed. Participants are encouraged to consult with the instructor individually before and after class about problems or difficulties they encounter in the class.

### 2.3 Fatty acid analysis

Fatty acids were isolated from plasma after total lipid extraction and derivatization. 50 μl of plasma was spiked with 5 μl of a mixture of 980 μM 1,2,3-tripalmitoyl-d31 glycerol; 949 μM 1,2-distearoylphosphatidylcholine-d70; 1,090 μM 22:1(13Z) cholesterol ester and 1,368 μM 13Z,16Z,19Z-docosatrienoic acid in 1:1 toluene:methanol. The lipids were extracted by the addition of 407.5 μl isopropanol and 520 μl cyclohexane followed by vortexing. To continue the initial extraction, 463 μl of LC/MS grade water and 57 μl 1M ammonium acetate were added as per the procedures of Smedes (22). Samples were allowed to centrifuge for 5 min at room temperature at 2,000 × g. The top organic layer was transferred to a new vial and then 520 μl of cyclohexane was added to the remaining aqueous phase to initiate the second extraction. Samples were centrifuged a second time under the same conditions and the remaining organic phase was extracted and added to the organic phase from the first extraction. Samples were then reduced to dryness under vacuum and reconstituted in 200 μl of 1:1 toluene:methanol. Derivatization of the fatty acids was performed using 20 μl aliquots of the extract. To the extract, 20 μl of 1:1 methanol:toluene, 20 μl of 60 μM of 10Z-pentadecenoic acid in methanol, 140 μl of methanol, and 100 μl of 0.5M sodium methoxide were added and vortexed and then allowed to incubate at 60°C for 1 hour.

After cooling, 100 μl of methanolic hydrochloric acid was added and samples were incubated at 60°C for 30 min. To begin back extraction, 400 μl of neutralizing solution (0.25M KHCO_3_, 0.5M K_2_CO_3_) and 400 μl of hexanes were added, samples were vortexed and centrifuged at 2,000 × g at room temperature for 5 min. To a new vial, 100 μl of the upper hexane layer of the samples and 10 μl of internal standard solution (44 μM C23:0 in hexane) were added and vortexed. The fatty acid methyl esters (FAMEs) were separated by GC/MS on a 7,890 GC with a 30m x 0.25mm, 0.25 μm DB-225ms column and detected with a 5977B mass selective detector (Agilent Technologies, Santa Clara, CA). Spectral data was acquired in simultaneous selected ion monitoring/ full scan (SIM/Scan) mode using the following parameters: Scan 50–400m/z; SIM, Group 1: (2–45min): 55.1, 67.1, 74.1, 79.1, 368.1m/z. Calibrants and internal standards were purchased from Nu Chek Prep (Elysian, MN) or Sigma-Aldrich (St. Louis, MO). Data was acquired and processed with MassHunter (Agilent Technologies, Santa Clara, CA). FAMEs were quantified against an 8-point calibration curve and results expressed in mol % abundance.

### 2.4 Oxylipin and endocannabinoid/endocannabinoid-like compound measurements in plasma

#### 2.4.1 Oxylipin and endocannabinoid/endocannabinoid-like compound extraction

Target analytes were isolated by solid phase extraction (SPE) on 10 mg Oasis-HLB sorbent beds from Waters (Milford, MA) in a 96 well plate format in the presence of deuterated internal standards. Prior to extraction, SPE wells were washed with 1 ml ethyl acetate followed by 2x 1 ml methanol and conditioned with 2x 1 ml of water/methanol 95:5 (v/v) with 0.1% acetic acid. Plasma samples (50 μl) were premixed in a 2 ml deep-well 96 well plate, with 5 μl of 0.2 mg/ml solution BHT/EDTA in 1:1 MeOH:H_2_O and 10 μl of a 20-compound surrogate mix of deuterium labeled oxylipins, acylethanolamides, acylglycines, monoacylglycerols, and polyunsaturated fatty acids in methanol to achieve final concentrations listed within Supplemental Table 4. Samples were then diluted with 1 ml of water/methanol 95:5 (v/v) with 0.1% acetic acid, transferred to the SPE plate and eluted by gravity. After a washing step with 1 ml of water/methanol 70:30 (v/v) with 0.1% acetic acid, sorbent beds were dried by vacuum at 7.5in Hg for 20 min. Samples were then eluted with 0.25 ml MeOH with 1% acetic acid, followed by 1 ml ethyl acetate into a deep-well 96 well plate containing 10 μl of 20% glycerol solution in MeOH in each well. Solvents were evaporated using a Genevac Ez2-Plus (SP Scientific, Warminster, PA) and residues were re-constituted with 250 μl of 1:1 MeOH/acetonitrile containing 100 nM 1-cyclohexyl ureido, 3-dodecanoic acid (CUDA) and 1-phenyl ureido 3-hexanoic acid (PUHA) (Cayman Chemical; Ann Arbor, MI). Samples were vortexed for 1 min to dissolve residues, chilled 15 min at 20 °C and filtered with a 0.2 μm PVDF centrifugal filter plate (Agilent Technologies).

#### 2.4.2 Sample analysis and quantification

Analytes were separated by ultra-performance liquid chromatography on a Nexera X2 (Shimadzu, Japan) using a 150mm x 2.1mm, 1.7μm Acquity BEH C18 column (Waters Corp, Milford, MA) and quantified on a API 6,500 QTRAP (SCIEX, Framingham, MA) according to previously described methods (23). Analytes were quantified using isotope dilution and internal standard methodology with 5 to 7 point calibration curves. Data was processed using MultiQuant v 3.2 (SCIEX). A complete list of analytical parameters are provided in Supplemental Tables 1–4.

#### 2.4.3 Data quality and reporting

Method performance was evaluated using procedural blanks, sample replicates, surrogate recovery standards, and monitoring of d5-2-AG isomerization. Analytical performance was considered acceptable based on surrogate recoveries, replicate precision, and the stability of the d5-2-AG internal standard throughout sample preparation and analysis. Mean surrogate recovery was 65% (range: 19–111%), with only one analyte (d8-C20:4n6) exhibiting an estimated recovery below 30%. Analytical precision, assessed from three duplicate sample pairs, was < 30% coefficient of variation for more than 65% of analytes present at concentrations >1 nM.

All plasma samples were collected, processed, stored, and analyzed using the same standardized procedures, thereby minimizing the potential for systematic bias arising from pre-analytical variability. Samples were maintained under controlled conditions, stored at −80 °C, and subjected to minimal freeze–thaw cycles prior to analysis. Assessment of d5-2-AG isomer stability indicated ~5 ± 6% isomerization (i.e. 1-AG formation) during extraction and instrumental analysis as shown in Supplemental Figure 1. Furthermore, no evidence of substantial *ex-vivo* lipid generation was observed during sample handling and processing. Results are reported in nM for analytes with available authentic standards. For analytes lacking commercially available standards (designated as screened analytes), results are reported as relative abundance across all measured samples, with the summed abundance for each metabolite normalized to 100%. Overall, analytical results met predefined quality-control criteria with respect to surrogate recoveries, replicate precision, and d5-2-AG isomer stability. Of the 130 analytes included in the analytical panel, approximately 30% were detected and fulfilled all quality-control requirements, whereas 47% were not detected in the analyzed samples.

### 2.5 Statistical analyses

All statistical analyses were performed in Jmp Student Edition 18.2.1 (SAS Institute, Clary NC). Partial least squares discriminant analysis highlighted changes between pre- and post-intervention samples. Paired *t*-tests with Benjamini and Hochberg false discovery rate corrections (24) were performed to examine the differences in plasma OxLs and eCBs of PM women (58.9 ± 5.2 years old, BMI: 33.6 ± 5.4 kg/m) completed the study.

## 3 Results

### 3.1 Tai Chi

All subjects successfully completed the four sessions of TC in the allotted time for the study duration.

### 3.2 Plasma fatty acids

Values for fatty acids are presented quantitatively as nmol/ml of plasma shown in Table 2. Values between pre- and post-TC did not change in the subjects (Table 2). All subjects had adequate levels of essential n-6 and n-3 PUFA and longer chain PUFA in plasma.

**Table 2:** Total plasma fatty acids in plasma (nmol/ml) of postmenopausal women subject to short–term Tai Chi.

Fatty acid	Pre (Mean±SD)	Post (Mean±SD)	*t*	P value
12:0	42.6 ± 45.9	26.2 ± 20.1	1.21	0.3
14:0	143.7 ± 74.3	157 ± 100	−0.50	0.6
15:0	28.1 ±10.2	30.0 ± 10.4	−0.61	0.6
16:0	3,460 ±1,100	3,270 ± 1,220	0.56	0.6
17:0	30.4 ± 9.5	29.3 ± 9.3	0.47	0.6
18:0	1,040 ± 322	996 ± 282	0.48	0.6
19:0	1.65 ± 0.87	1.57 ± 0.62	0.41	0.7
20:0	4.71 ± 3.36	4.51 ± 2.39	0.25	0.8
21:0	0.430 ± 0.310	0.210 ± 0.270	0.35	0.8
22:0	2.46 ± 2.93	2.82 ± 2.97	−0.35	0.7
24:0	2.12 ± 1.49	2.13 ± 1.67	−0.02	1
14:1n5	4.73 ± 3.34	6.52 ± 5.05	−1.01	0.3
16:1n7t	22.0 ± 15.6	23.6 ± 19.7	−0.37	0.7
16:1n7	264 ± 122	241 ± 130	0.59	0.6
17:1n7	1.94 ± 0.00	0.620 ± 0.560	–	–
18:1n9	3,000 ± 1,160	2,880 ± 1,320	0.32	0.8
18:1n7	192 ± 42	181 ± 46	0.88	0.4
19:1n9	2.58 ± 2.38	1.41 ± 0.99	1.16	0.3
20:1n9	5.56 ± 3.77	4.72 ± 4.96	−0.12	0.9
24:1n9	18.9 ± 20.6	ND	–	–
18:2n6	4,410 ± 1,810	4,430 ± 1,600	−0.05	1
18:3n6	53.5 ± 23.9	64.1 ± 30.9	−1.17	0.3
9c,11t–CLA	12.4 ± 5.4	11.2 ± 5.6	0.81	0.4
10t,12c–CLA	1.16 ± 0.72	0.840 ± 0.300	1.65	0.1
20:3n6	176 ± 62	173 ± 62.2	0.20	0.8
20:4n6	893 ± 261	855 ± 216	0.56	0.6
22:2n6	1.03 ± 1.01	0.580 ± 0.370	1.43	0.2
22:4n6	60.2 ± 53.9	19.9 ± 3.13	–	–
22:5n6	20.6 ± 12.9	7.82 ± 5.55	0.41	0.8
18:3n3	89.6 ± 73.6	98.6 ± 64.0	−0.51	0.6
18:4n3	2.99 ± 1.22	3.86 ± 3.39	−0.89	0.4
20:3n3	3.85 ± 4.64	2.49 ± 2.69	1.01	0.4
20:4n3	5.77 ± 7.25	6.10 ± 6.10	−0.56	0.6
20:5n3	42.6 ± 34.3	43.8 ± 31.1	−0.23	0.8
22:5n3	44.0 ± 10.6	41.7 ± 15.4	0.60	0.6
22:6n3	172 ± 79.2	162 ± 81.5	0.48	0.6
Σ SFA	4,750 ± 1,480	4,510 ± 1,610	0.52	0.6
Σ MUFA	3,480 ± 1,300	3,340 ± 1,500	0.37	0.7
Σ (n−6) PUFA	5,580 ± 1,850	5,540 ± 1,710	0.11	0.9
Σ (n−3) PUFA	356 ± 130	354 ± 155	0.03	1
Total fatty acids	14,200 ± 4,570	13,700 ± 4,770	0.35	0.7

Values from 16 subjects. ND = not detected. No t-test or P value where one value was found.

### 3.3 Plasma eCBs and OxLs

**Figure 1:**
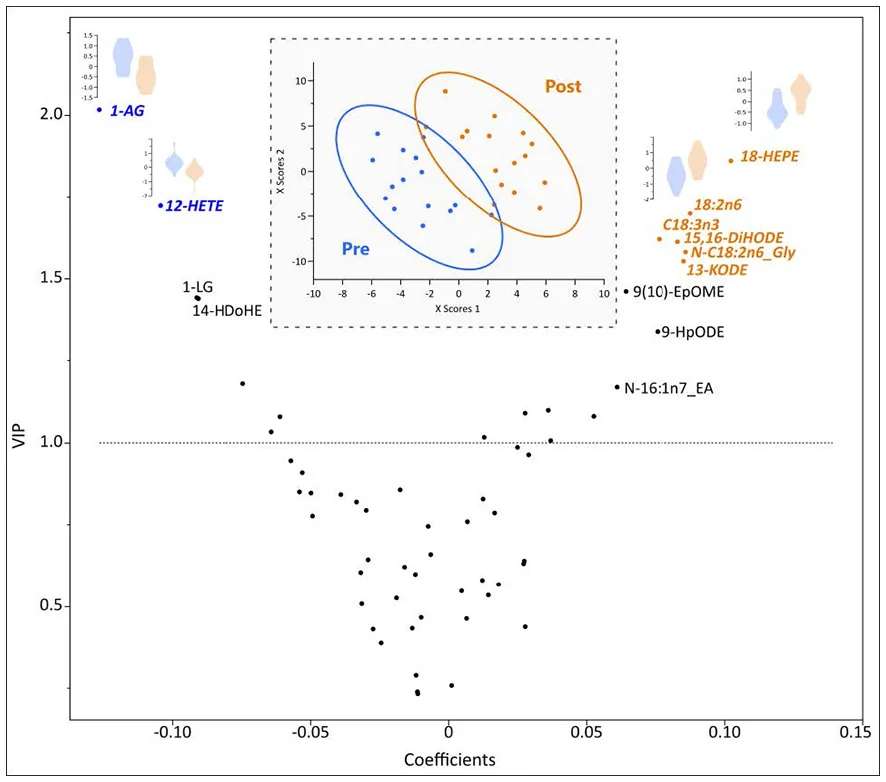
Partial Least Squares Discriminant analysis loading plot, with inset scores plot, of metabolite residuals adjusting for subject as a random effect. Metabolites are labeled if significantly different and passing false discovery rate at q ≤ 0.05 (bold italic) or q ≤ 0.1. Blue = Pre; Orange = Post. The best fit model explained 65% of the system variance with 4 latent variable (Q2 = 0.992; R2 X = 0.660; R2 Y = 0.953). The first 2 latent variables plotted here explained 50% of the variance. The time group least squared means for the top 4 metabolites are shown as violin plots, accounting for subject as a random effect. VIP = variable importance in projection.

The partial least square discriminate analysis of subjects using plasma eCBs and OxLs concentrations yielded a good discriminating model with 4 latent variables (Q2 = 0.992; R2 X = 0.660; R2 Y = 0.953) explaining 65% of the total variance. The resulting score and loading plots are shown in Figure 1. Interestingly, after the 4th TC session compared to baseline, several OxL values showed important PLS scores greater than 1.0 (Figure 1). Further, the pre- and post-sessions values revealed a shift toward positive X Scores post-sessions. The X scores in PLS regression summarize the original X variables and are used to predict Y. Generally, 1-AG and 12-hydroxyeicosanoic acid (12-HETE) were lower, and 18-hydroxypentaenoic acid (18-HEPE) and N-linoleoyl glycine (N-18:2n6 Gly) higher post-sessions (Table 3), with PLS variable importance in projection scores >1.0 (Figure 1). While not significantly altered, plasma concentrations of the EPA-derived 12-HEPE and DHA derived 14-HDoHE were strongly correlated with 12-HETE, with *r* = 0.73 (*p* < 0.0001) and *r* =0.832 (*p* < 0.0001). Moreover, the autooxidation products 9-HETE and F2-isoprostanes were not detected.

**Table 3:** Plasma lipid mediator and nonsteroidal anti-inflammatory concentrations in postmenopausal women subject to short-term Tai Chi.

OxL or eCB	Units	Pre (Mean ±SD)	Post (Mean ±SD)	P value
9–HpODE_(screen)	% Rel Ab	1.79 ± 1.60	4.07 ± 5.32	0.08
13–HpODE_(screen)	% Rel Ab	2.69 ± 2.38	3.18 ± 4.90	1
15–HETE	nM	2.15 ± 1.10	2.35 ± 1.71	1
**12–HETE**	**nM**	**4.93** **±1.15**	**4.31** **±1.44**	**0.0256**
**18–HEPE**	**nM**	**0.39** **±0.35**	**0.750** **±1.12**	**0.00890**
12–HEPE	nM	4.31 ± 1.15	4.93 ± 1.44	0.5
14–HDoHE	nM	2.79 ± 3.08	2.06 ± 2.73	0.08
9(10)–EpO	nM	57.6 ± 38.5	63.9 ± 26.6	0.6
12(13)–EpOME	nM	41.6 ± 27.2	48.6 ± 22.4	0.5
9(10)–EpOME	nM	22.6 ± 16.1	32.0 ± 12.4	0.07
15(16)–EpODE_(screen)	% Rel Ab	2.81 ± 2.88	3.06 ± 2.47	0.7
9(10)–EpODE_(screen)	% Rel Ab	2.53 ± 1.68	3.32 ± 2.16	0.4
12(13)–EpODE_(screen)	% Rel Ab	2.44 ± 1.58	3.35 ± 2.06	0.3
14(15)–EpETrE	nM	2.02 ± 1.47	1.78 ± 0.75	1
11(12)–EpETrE	nM	0.850 ± 0.760	0.850 ± 0.700	1
19(20)–EpDoPE	nM	1.81 ± 1.20	2.41 ± 4.33	1
9,10–DiHO	nM	22.5 ± 13.7	26.9 ± 14.1	0.6
12,13–DiHOME	nM	11.7 ± 8.3	12.6 ± 7.5	0.7
9,10–DiHOME	nM	9.21 ± 8.21	11.9 ± 10.6	0.7
**15,16–DiHODE_(screen)**	**% Rel Ab**	**2.20** **±1.48**	**3.66** **±2.43**	**0.0261**
12,13–DiHODE_(screen)	% Rel Ab	2.65 ± 3.26	3.06 ± 2.88	0.8
9,10–DiHODE_(screen)	% Rel Ab	2.76 ± 3.12	3.13 ± 3.37	0.8
14,15–DiHETrE	nM	0.620 ± 0.310	0.620 ± 0.250	0.9
11,12–DiHETrE	nM	0.270 ± 0.130	0.230 ± 0.100	0.6
8,9–DiHETrE	nM	0.240 ± 0.200	0.240 ± 0.210	0.8
17,18–DiHETE	nM	0.263 ± 0.158	0.265 ± 0.192	1
19,20–DiHDoPE	nM	1.98 ± 1.46	1.85 ± 2.32	0.6
16,17–DiHDoPE	nM	0.130 ± 0.110	0.140 ± 0.140	0.9
2–AG	nM	11.5 ± 5.2	12.2 ± 17.8	0.2
**1–AG**	**nM**	**7.77** **±4.14**	**4.46** **±3.21**	**0.00890**
2–LG	nM	261 ± 146	277 ± 350	0.5
1–LG	nM	292 ± 167	218 ± 264	0.09
2–OG	nM	410 ± 232	454 ± 357	1
1–OG	nM	454 ± 264	586 ± 980	0.8
N–C16:0_EA	nM	15.5 ± 4.8	17.7 ± 7.1	0.6
N−16:1n7_EA	nM	0.690 ± 0.360	0.980 ± 0.590	0.2
N–C18:1n9_EA	nM	4.36 ± 2.34	4.22 ± 2.37	1
N–C20:4n6_EA	nM	1.88 ± 1.40	1.45 ± 0.70	0.6
N–C18:1n9_Gly	nM	7.73 ± 4.28	8.12 ± 5.90	0.9
**N–C18:2n6_Gly**	**nM**	**2.28** **±0.94**	**2.81** **±0.82**	**0.0261**
Naproxen^*^	nM	11,700 ± 37,800	11,600 ± 46,000	0.8

Values from 16 subjects are mean and standard deviation of plasma in subjects pre– and post-TC of 4 sessions. P-values are pre-post-comparisons, controlling for subject as a random effect and FDR adjusted. ^*^ Naproxen and ibuprofen use were opportunistically determined in the targeted metabolomics panel to allow for statistical adjustment in COX-dependent metabolites. Elevated Naproxen was observed in 2 (2-Pre, 1-Post TC) participants. Ibuprofen was elevated in a single participant at baseline (data not shown).

Four sessions of TC in PM women showed other yet minor effects on OxLs and eCBs from the *t*-test (Table 3). After TC the level of AEA was not changed. There was a significant decline in 1-AG but no observable change in 2-AG after TC. Effects on many OxLs highlighted by the PLS-DA analysis such as higher levels of 14-HDoHE, 9(10)-EpOME, and 5,16-DiHODE were not significant at P ≥ 0.05 by FDR adjusted *t*-tests (Table 3). TC was of short duration and modestly affected eCB; however, it is not clear if endocannabinoid tone and eCB receptor (type 1 and type 2) changes can influence peripheral tissues (3) in short term TC exercise. Moreover, it is interesting that a higher level of N-C18:2n6 Gly was found after TC which might reflect an exercise effect for supporting cellular signaling (23).

## 4 Discussion

We found differences in plasma OxL concentrations in short term TC exercise non-fasted female subjects. However, we did not examine metabolic or physiologic exerkines (8) which are implicated in exercise and pain (14, 25). We previously reported that TC exercise is an approach to minimize pain and improve brain plasticity associated with changes in AEA and OxLs in individuals suffering from arthritic pain (18). In the present study of individuals, after TC plasma concentrations of 12-HETE and 1-AG were lower, while 18-HEPE, 15,16-DiHODE, N-18:2n6 Gly linoleate and alpha-linolenate were higher. OxLs constitute a superclass of compounds formed biosynthetically in mammals via cyclooxygenase (COX), lipoxygenase (LOX), and cytochrome P450 (CYP) enzymes and through interactions with reactive oxiygen (26). Notably the balance of these metabolites are also influenced by the availability of their precursor PUFAs (17). While 12-HETE is often associated with platelet function, and platelet degranulation can artifactually elevate plasma 12-HETE levels, leukocytes vascular endothelium and smooth muscle, as well as adipose tissue are also capable of producing this metabolite (27, 28). While it is unclear what drives the changes in 12-LOX products (i.e. 12-HETE, 14-HDoHE) in the post TC treatment, the lack of measurable thromboxane argues against its generation by activated platelets (29). Moreover, the strong correlation of 12-HETE, 14-HDoHE, and 12-HEPE, with weaker correlations to 15-HETE and a lack measurable of 9-HETE and F2-isoprostanes argues for a 12-lipoxygenase, rather than an autoxidative source (30–32). Regardless, the reduction in plasma concentrations of 12-HETE with the increase in 18-HEPE and N_18:2n6 Gly concentrations does support a shift toward an anti-inflammatory state (33–35) and possibly improved insulin sensitivity (27).

The subjects consumed adequate amounts of essential omega-3 and omega-6 PUFA based on the fatty acid analysis of plasma. However, because most OxLs are produced by these essential PUFA, dietary status of both n-3 and n-6 PUFA can alter the amounts and types of the OxLs and other lipid mediators including endocannabinoids and endocannabinoid-like compounds (36, 37). 12-HETE is biosynthesized from arachidonic acid an n-6 PUFA and 12-HEPE from eicosapentaenoic acid an n-3 PUFA. The former is generally proinflammatory and pro-thrombotic whereas the latter is less inflammatory and affords metabolic benefits (34, 38). In addition, 15,16-DiHODE (15,16-dihydroxy-9,12-octadecadienoic acid) is the soluble epoxide hydrolase product of 15(16)-EpODE (15(16)-epoxy-9,12-octadecadienoic acid) derived from alpha-linolenic acid (ALA) by CYP metabolism (39). While often associated with the broader, generally proinflammatory category of dihydroxyoctadecadienoic acids (DiHOMEs/DiHODEs), 15,16-DiHODE is recognized as a significant biomarker and mediator in inflammatory processes and may be associated with inflammation process (26, 39).

Monoacylglycerols are marginally stable in aqueous environments, with the enzymatically derived 2-acylglycerols spontaneously isomerizing with acyl-migration to the thermodynamically more stable 1-acylglycerol isomers (20) which if allowed to reach equilibrium will obtain a 9:1 sn-1 MAG:sn-2 ratio (40). In the current data set, the 1-AG:2-AG ratio was 0.6 ± 0.2, suggesting that while likely, acyl migration was limited. Importantly, the 1- (and 3-) AGs are more rapidly degraded than the 2-AG and have reduced potency of interaction with cannabinoid receptors (41). While we have no direct measures of lipase activity, lower plasma concentrations of 1-AG [and 1-LG (p = 0.08)] without changes in 2-acylglycerols are consistent with the increased lipolytic clearance of the 1-vs. 2-acylglycerols. Although the 2-AG is the more active isomer, 1-AG stimulates CB-1 Ca^+2^ signaling *in vitro* (42). Moreover, the transformation of 2-AG into 1-AG appears to be an important stabilizer and influencer of the strength of 2-AG action (42). While the total circulating MAG concentrations were not altered by TC exercise, the preferential depletion of the 1-acyl isomers does suggest an increased lipolytic clearance during physical activity (19).

The ECS is widely distributed throughout peripheral tissues, including skeletal muscle, adipose tissue, liver, and immune cells; however, the expression and functional roles of ECS components vary considerably among tissues (43). Skeletal muscle expresses cannabinoid receptors, endocannabinoid metabolic enzymes, and endogenous ligands, with CB1 receptor expression generally exceeding that of CB2 (44, 45). In contrast, adipose tissue exhibits robust ECS activity and is considered an important contributor to systemic endocannabinoid production, energy homeostasis, and metabolic regulation (43). Similarly, oxylipin biosynthesis and signaling are tissue-dependent, reflecting differences in fatty acid availability, enzymatic pathways, and local inflammatory status (46). In the present study, due to funding limitations it was not possible to conduct a comprehensive analysis of the enzymes and receptors of eCB in muscle and adipose. However, with this new data additional research is justified on the biology of eCB and OxL given that apparent shift in monoacylglycerols and oxylipin concentrations likely represents the integrated output of multiple tissues rather than the activity of a single organ system (43).

The interpretation of circulating eCB and OxL profiles should consider the tissue-specific nature of their production and signaling. Although exercise-induced changes in plasma metabolites are often discussed in the context of skeletal muscle adaptations, endocannabinoids and oxylipins may originate from multiple tissues, including adipose tissue, immune cells, vascular tissues, and the liver. Differences in cannabinoid receptor expression, metabolic enzyme abundance, and lipid signaling pathways across tissues may influence both the magnitude and physiological significance of circulating biomarker responses. Therefore, the plasma concentrations measured in the present study should be viewed as systemic indicators of ECS and lipid mediator activity rather than direct measures of tissue-specific responses. Future studies incorporating tissue sampling or targeted mechanistic approaches will be important for identifying the specific organ systems contributing to the observed circulating metabolite profiles.

A limitation of this study is that blood samples were collected in a non-fasted state. Nutritional status and recent food intake can influence circulating concentrations of both eCB and OxL because these lipid mediators are derived from dietary and tissue PUFA and exhibit postprandial responses. Previous studies have demonstrated that circulating OxL and eCB profiles can change following meal consumption, particularly when dietary fatty acid composition is altered, and that some metabolites show greater variability in the postprandial state than under fasting conditions. In addition, dietary fatty acid intake is a recognized determinant of oxylipin production and circulating OxL patterns. Consequently, some of the variability observed in this study may have been influenced by differences in participants' recent dietary intake. However, because blood samples were collected under similar non-fasting conditions at both baseline and post-intervention assessments, the within-subject pre–post design likely reduced, although did not eliminate, the potential confounding effects of feeding status. Future studies should incorporate standardized fasting conditions or controlled pre-sampling meals to better isolate the effects of Tai Chi on circulating eCB and OxL responses (47, 48). Moreover, since isomer specific changes in monoacylglycerides were observed aggressive pre-analytical steps will be needed in the future to suppress acyl-migration, to better understand Tai Chi impacts on endogenous relative isomeric abundance and concentrations. Such steps may include immediate blood processing, the addition of the lipase inhibitor Orlistat to EDTA blood collections (20), and/or the immediate acidification of collected blood to pH 4.7 (49). It should be noted that simultaneous considerations for oxylipins stability should be considered (50). Finally, because achiral chromatographic procedures were used, it should be emphasized that the reported 1-AG concentrations may be mixtures of the 1(S)- and 3(R)-monoacylglycerols.

## 5 Conclusion

We report that short-term 4 TC sessions had less impact on eCBs and OxLs in self-reported PM women compared to our previous 8-week TC exercise study in PM women with knee osteoarthritis (18, 19). The observed changes in generally healthy women are consistent with an anti-inflammatory shift in metabolism but no profound impacts on the eCBs recognized to improve mood with physical exercise (51, 52) and after TC exercise (18, 19). New directions for the research should include clinical trials of longer TC intervention and a larger sample size in PM women to confirm the amount of time needed and assess the dependence on the acute arthritic pathophysiology for changes in eCBs and OxLs we previously reported (18, 19). Future studies should include measurements of enzyme expression for eCB (phospholipase C, DAGL-alpha, MGL, FAAH) and those of the LOX pathways. Future studies should also investigate the tissue-specific expression and localization of enzymes involved in eCB metabolism to better identify the biological sources and regulatory mechanisms underlying changes in circulating eCBs following TC. Such investigations would include different dietary PUFA (n-6 and n-3) that serve as eCB and OxL substrates. Future studies should also measure circulating free fatty acids, as exercise-induced fatty acid mobilization may contribute to the generation of oxylipins and help elucidate the mechanisms underlying TC-associated changes in lipid mediator profiles. Studies with self-reported males must be conducted to identify sex differences during TC. New studies should evaluate if TC changes blood metabolic and physiologic exerkines such as lactate and myokines to better understand exercise effects on general health and brain plasticity changes associated with exercise (8).

## Data Availability

The original contributions presented in the study are included in the article/Supplementary material, further inquiries can be directed to the corresponding authors.
