# Supplementary material for: Tai Chi exercise of short duration alters circulating oxylipins in postmenopausal women: a pilot study
Source: Front Med (Lausanne). 2026 Aug 31;13:1907924. doi: 10.3389/fmed.2026.1907924 (PMC13572237; doi:10.3389/fmed.2026.1907924)
Supplement: Supplementary file 1 [file Data_Sheet_2.docx]

**Supplemental Fig 1: Documentation of arachidonylglycerol isomerization and isomer abundance in analyzed plasma samples. A)** Deuterated 2-arachidonoyl glycerol (d5 2-AG) levels in calibration standards and a representative sample are shown. The d5-2-AG solution used shows ~10% isomerization at the time of use. Samples showed an average of 5% greater d5 1-AG than calibration solutions (p <0.001) suggesting significant, yet minimal isomerization during sample processing. **B)** Relative abundance of native 2-AG and 1-AG in level 3 calibration standard (1:1 concentration), National Institues of Technology Standard Reference Material 1950, Metabolites in Human Plasma (NIST SRM 1950), and a representative sample. We consider NIST SRM 1950 to represent maximally isomerized plasma monoacylglycerols due to the procedures used to prepare this material.
